# Association between the retinal vascular network with Singapore "I" Vessel Assessment (SIVA) software, cardiovascular history and risk factors in the elderly: The Montrachet study, population-based study

**DOI:** 10.1371/journal.pone.0194694

**Published:** 2018-04-03

**Authors:** Louis Arnould, Christine Binquet, Charles Guenancia, Seydou Alassane, Ryo Kawasaki, Vincent Daien, Christophe Tzourio, Yumiko Kawasaki, Abderrahmane Bourredjem, Alain Bron, Catherine Creuzot-Garcher

**Affiliations:** 1 Department of Ophthalmology, University Hospital, Dijon, France; 2 INSERM, CIC1432, clinical epidemiology unit, Dijon, France, Dijon University Hospital, Clinical investigation Center, Clinical epidemiology/clinical trials unit, Dijon, France; 3 Department of Cardiology, Dijon University Hospital, Dijon, France; 4 INSERM, UMR866, Dijon University Hospital, Laboratory of Cardiometabolic Physiopathology and Pharmacology, Dijon, France; 5 Eye and Nutrition Research group, CSGA, UMR 1324 INRA, Dijon, France; 6 Department of Public Health, Yamagata University Graduate School of Medical Science, Yamagata, Japan; 7 Department of Ophthalmology, University Hospital, Montpellier, France; 8 INSERM, U708, Epidemiology, University Hospital, Bordeaux, France; Institut de la vision, FRANCE

## Abstract

**Purpose:**

To identify patterns summarizing the retinal vascular network in the elderly and to investigate the relationship of these vascular patterns with cardiovascular history.

**Methods:**

We conducted a population-based study, the Montrachet study (Maculopathy Optic Nerve nuTRition neurovAsCular and HEarT diseases), in participants older than 75 years. The history of cardiovascular disease and a score-based estimation of their 10-year risk of cardiovascular mortality (Heart SCORE) were collected. Retinal vascular network analysis was performed by means of Singapore “I” Vessel Assessment (SIVA) software. Principal component analysis was used to condense the information contained in the high number of variables provided and to identify independent retinal vascular patterns.

**Results:**

Overall, 1069 photographs (1069 participants) were reviewed with SIVA software. The mean age was 80.0 ± 3.8 years. We extracted three vascular patterns summarizing 41.3% of the vascular information. The most clinically relevant pattern, *Sparse vascular network*, accounted for 17.4% of the total variance. It corresponded to a lower density in the vascular network and higher variability in vessel width. Diabetic participants with hypoglycemic treatment had a sparser vascular network pattern than subjects without such treatment (odds ratio, [OR], 1.68; 95% CI, 1.04–2.72; *P* = 0.04). Participants with no history of cardiovascular disease who had a sparser vascular network were associated with a higher Heart SCORE (OR, 1.76; 95% CI, 1.08–2.25; *P* = 0.02).

**Conclusions:**

Three vascular patterns were identified. The *Sparse vascular network pattern* was associated with having a higher risk profile for cardiovascular mortality risk at 10 years.

## Introduction

Demographic indicators confirm that life expectancy in industrialized countries has increased over the last few decades. However, an estimated 17.5 million people worldwide died from cardiovascular diseases in 2012, accounting for 31% of all deaths worldwide [1]. Although cardiovascular case fatality decreased in high-income countries, recurrent cardiovascular disease (CVD) events are common [2]. Moreover, a study in the US showed that CVDs were one of the major medical reasons for rehospitalization, with a rate ranging from 14.5% to 26.9% [3]. CVDs have also increased in developing countries [4]. As a consequence, the burden of CVD remains a key priority globally. Since in most patients the underlying pathophysiological process of CVD develops long before their diagnosis, simple examinations to detect early vascular remodeling would be useful for early identification of high-risk subjects. Previous studies have suggested that microcirculatory changes are closely related to cardiovascular modifications in humans [5,6].

Fundus photography provides an *in vivo* noninvasive view of the human microcirculation network [7]. Indeed, specific software programs have been developed to analyze the retinal vascular network automatically and provide a description of the geometric characteristics of its arterial and venous components. Particularly, the Singapore “I” Vessel Assessment (SIVA) software was proved to be highly accurate and reproducible in assessing multiple *in vivo* architectural changes in the retinal vascular network [8,9]. These improvements may allow considering fundus photography to be a suitable exam to assess the vascular aging process in the general population. Several recent studies focused on a single geometric feature of the retinal vascular network. Significant associations were found, notably between retinal vasculature alteration and risk of hypertension or coronary heart disease mortality, but only a few microvascular characteristics were investigated [10,11]. Nevertheless, automatized image analysis allows dozens of geometric features to be interpreted. A thorough description without an *a priori* selection of retinal vascular features is needed to establish a better understanding of which of these features or combinations of these features are the most promising candidates to become a biomarker of history of CVD.

The purpose of this study was first to identify retinal vascular patterns in the elderly within a population-based study. Then we sought to determine the associations between these vascular patterns, cardiovascular history and the 10-year risk of fatal CVD in this population.

## Methods

The Montrachet (Maculopathy Optic Nerve nuTRition neurovAsCular and HEarT diseases) Study focused on participants older than 75 years. This population-based study was designed to assess the associations between age-related eye diseases and neurologic and heart diseases in the elderly [12]. Participants in the Montrachet study were recruited from an ongoing population-based study, the Three-City (3C) study. The 3C study was designed to examine the relationship between vascular diseases and dementia in 9294 persons aged 65 years and over [13]. The participants were selected from the electoral rolls in an urban setting, all living in three French cities: Bordeaux, Dijon and Montpellier. At baseline and every 2 years, the participants filled in a complete questionnaire on their cardiovascular (myocardial infarction, angina, coronary artery dilatation, coronary bypass and cardiovascular morbidity) and neurological history (ischemic and hemorrhagic stroke) as well as their treatments. Blood pressure, weight and height were measured and a blood sample (lipid, blood glucose tests and creatinine values) was collected after fasting.

In Dijon, 4931 individuals participated in the first run of the 3C Study in 1999. At the fifth run (10 years later), a subgroup of participants was invited to participate in the Montrachet Study. The study was approved by Dijon University Hospital ethics committee and was registered as 2009-A00448–49. All participants gave their informed consent, and the study followed the tenets of the Declaration of Helsinki. Each participant had a complete eye examination including fundus photography in the Department of Ophthalmology, Dijon University Hospital, Dijon, France [12]. Forty-five-degree color retinal photographs, centered on the optic disc, were performed on both eyes with a fundus camera (TRC NW6S, Topcon, Tokyo, Japan) after pupil dilation with tropicamide 0.5% (Thea, Clermont-Ferrand, France). Fundus photographs acquired during the study were anonymously sent to the reading center in Yamagata University, Japan (RK and YK), and a single trained grader extracted retinal vessel characteristics with the Singapore “I” Vessel Assessment (SIVA) software. Retinal vascular network computerized analysis was based on the analysis of vessels from the center of the optic disc and then to three successive zones corresponding to 0.5 (zone A), 1 (zone B) and 2 (zone C) disc diameter. The six largest arterioles and veins were analyzed. Only one eye was retained for analysis and selection of fundus photographs followed the criteria described below: 1) fundus photograph of the right eye for participants born in even-numbered years and the left eye for those born in odd-numbered years; 2) in single-eye patients, the functional eye was selected; 3) when a picture was uninterpretable on one eye, the other one was retained for analysis.

A fundus photograph was considered uninterpretable if blurred, if zone C could not be analyzed or if the fundus photograph contained fewer than six arterioles and veins. Geometric retinal vascular features including fractal dimension, vascular caliber, tortuosity and branching angle were collected. They were measured by means of the semi-automated SIVA software. The grading system was based on algorithms for automated optic disc detection with automatic vascular structure extraction and tracing. Then retinal arterioles and venules were identified automatically. Finally, formulas and algorithms were used to compute quantifiable measurements of retinal vasculature.

### Statistical analysis

#### Pattern identification

The computerized analysis identified 54 geometric features [14–18]. To summarize these numerous data and to identify retinal vascular patterns, we performed a principal component analysis (PCA) based on these geometric characteristics. PCA extracts the most important information from a quantitative data set and reduces the number of variables by keeping only worthwhile information [19]. It builds up uncorrelated variables (components), which are linear combinations of a set of correlated variables (here geometric characteristics of the retinal vascular network). Coefficients defining these linear combinations, called factor loadings, may be interpreted as correlation coefficients. A positive (negative) factor loading means that the vascular feature is positively (negatively) associated with the component. An absolute value of loading close to 1 indicates a strong influence of the vascular feature on the value of the component. We used SAS ‘‘Varimax” orthogonal transformation to maximize the independence (orthogonality) of the factors retained and to obtain a simpler structure for easier interpretation. To determine the number of patterns to retain, we used Kaiser’s criterion (eigenvalues > 1), graphical analysis of the scree plot and the clinical interpretability of these factors [20–22]. The labeling of each pattern was primarily descriptive and based on our interpretation of the vascular features strongly associated with the component. We then calculated each patient’s score in relation to the component. The score indicates how well the patient fit the component. The fit corresponds to the distance between the patient and the component. We discretized these scores into tertiles (good, intermediate, poor fit).

#### Associations between cardiovascular background and each pattern

We assessed the associations between the patients’ characteristics (age, sex and education level), cardiovascular risk factors (smoking habits, body mass index [BMI], hypoglycemic treatment for diabetes mellitus, hypotensive treatment for hypertension and cholesterol-lowering treatment for dyslipidemia), cardiovascular and ischemic stroke history summarized in a single variable: major adverse cardiovascular and cerebrovascular events [MACCE]), and how well the patient fit each specific pattern identified in the preceding step. We first carried out a bivariate analysis using the chi-square test or the Fisher exact test, and the ANOVA test or the Kruskal-Wallis test when appropriate. Correlations between covariates were systematically checked to detect collinearity. Multivariate analysis was then performed using polytomous logistic regressions in which the dependent variable was how well the patient fit each retinal vascular pattern using tertiles. Age, gender and education level were forced in the model. Interactions between treated hypertension and diabetes were systematically tested because these two risk factors may interact with each other. The results were expressed as odds ratios and their 95% confidence intervals (95% CI).

In addition, the 10-year risk of fatal CVD was estimated by means of Montrachet baseline information using the method proposed in the Systematic COronary Risk Evaluation (Heart SCORE project). *The Heart SCORE is an age- and sex-specific risk chart that was developed based on cholesterol and systolic blood pressure levels and smoking habit*, *separately for high- and low-risk European populations*. We used the corresponding low-risk charts given the low risk observed in the French population [23]. Since the Heart SCORE risk charts are intended for risk stratification in primary prevention of cardiovascular disease, participants with previous MACCE were excluded. The association between each pattern fit and the 10-year risk of a fatal cardiovascular event was estimated using a polytomous logistic regression adjusted on age, education level, BMI and treated diabetes mellitus.

A P-value < 0.05 was considered significant. SAS software (version 9.4, SAS Institute, Inc., Cary, NC, USA) was used for all analyses.

## Results

**Fig 1:**
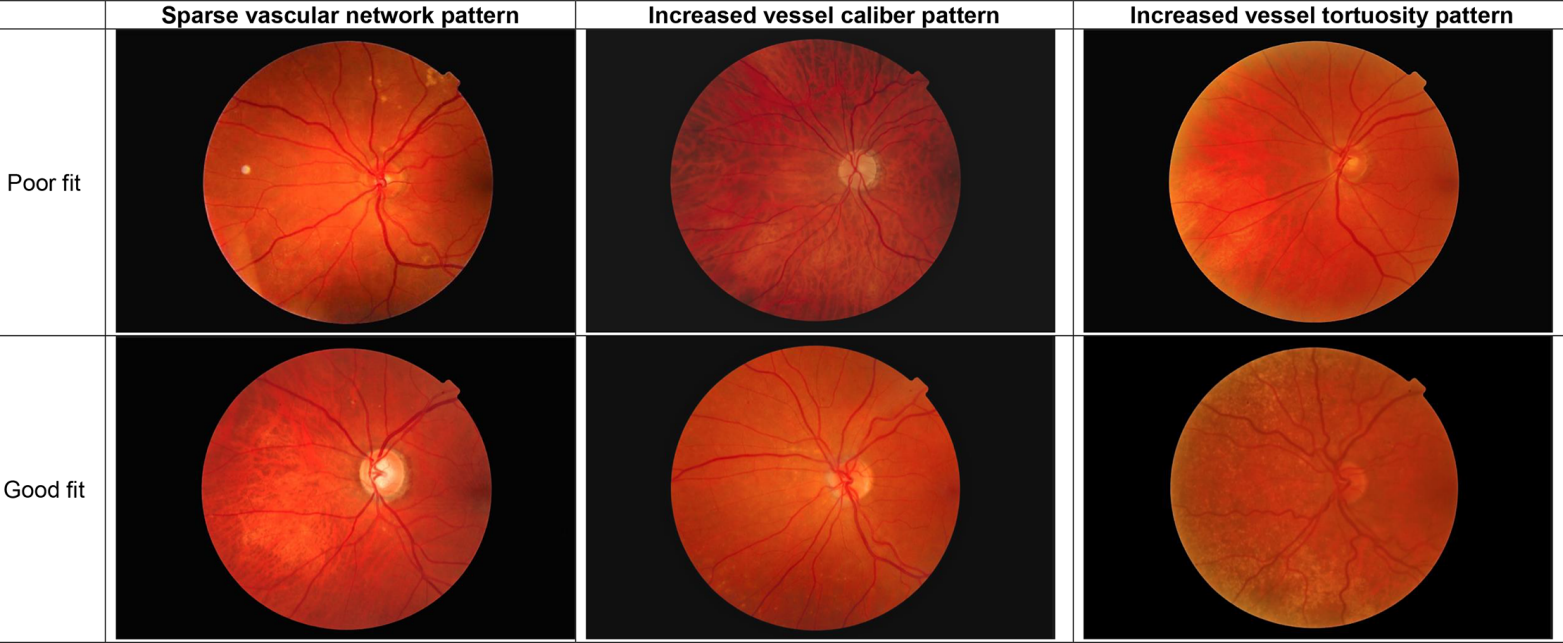
Poor and good fit for the 3 vascular patterns with SIVA software.

Among the 1153 subjects included in the Montrachet study, 1094 participants had interpretable fundus photography. After exclusion of subjects with epiretinal membrane (n = 25), 1069 retinal vascular network computerized analyses were performed. The characteristics of the study population and comparison between participants and nonparticipants are summarized in Table 1. No significant difference was found between subjects with fundus photography SIVA analysis and participants without SIVA analysis except for education level (P = 0.04). In the overall population, three vascular patterns were identified, which accounted for 41.3% of the total variance. The first pattern corresponded to lower density in the vascular network as well as greater variability in vascular width because it was mainly correlated with decreased fractal dimension and an increased vessel width standard deviation (Table 2). It accounted for 17.4% of the total variance. We named the first factor the *Sparse vascular network pattern*. The second pattern, the *Increased vessel caliber pattern*, was characterized by large vessel diameter and width; it accounted for 14.6% of the total variance. The third pattern showed increased tortuosity for both arterioles and venules. It accounted for 9.3% of the total variance. We named it the *Increased tortuosity pattern*. Fractal dimension, vessel caliber and tortuosity for each tertile and the related pattern are displayed in Table 3. Poor and good fit for the three patterns are presented in Fig 1. Table 4 and Table 5 show relationships between the subjects’ characteristics and these vascular patterns.

**Table 1:** Characteristics of participants with SIVA analysis at their inclusion in the Montrachet study (n) compared to participants (n′) without SIVA analysis.

	Participants with SIVA analysis (n = 1069)	Participants with no SIVA analysis (n’ = 84)	P-value
**Age, mean ± SD, y**	80.0 ± 3.8	80.5 ± 4.2	0.41
**Sex** (n = 1069 and n′ = 84)			0.14
Male	393 (36.8)	38 (45.2)	
Female	676 (63.2)	46 (54.8)	
**Education** (n = 1064 and n′ = 84)			**0.04**
Primary education	142 (13.4)	17 (20.2)	
Lower secondary education	467 (43.9)	25 (29.8)	
Upper secondary education	214 (20.1)	16 (19.0)	
Tertiary education	241 (22.6)	26 (31.0)	
**Smoking status** (n = 985 and n′ = 76)			0.29
Non-smokers	556 (57.5)	38 (50.0)	
Smokers	419 (42.5)	38 (50.0)	
**Treatment for systemic hypertension** (n = 1014 and n′ = 84)			0.89
No	360 (34.1)	28 (0.3)	
Yes for < 10 years	286 (28.2)	26(0.3)	
Yes for ≥ 10 years	382 (37.7)	31 (0.4)	
**Systolic blood pressure, mean in the 3C study ± SD, mmHg**	140.2 ± 16.8	138.3 ± 13.0	0.78
**Body mass index, mean in the 3C study ± SD, kg/m**^**2**^	25.5 ± 3.5	25.9 ± 3.6	0.85
**Hypoglycemic treatment** (n = 957 and n′ = 71)			0.26
No	836 (87.4)	64 (90.1)	
Yes for < 10 years	65 (6.8)	6 (8.5)	
Yes for ≥ 10 years	56 (5.8)	1 (1.4)	
**Cholesterol-lowering treatment** (n = 1021 and n′ = 80)			0.78
No	321 (31.4)	25 (31.3)	
Yes for < 10 years	187 (18.3)	17 (21.2)	
Yes for ≥ 10 years	513 (50.3)	38 (47.5)	
**Medical history at Montrachet inclusion**			
*CVD (n = 1018 and n′ = 84)*			0.40
No	943 (92.6)	75 (89.3)	
Yes < 10 years ago	51 (5.0)	7 (8.3)	
Yes ≥ 10 years ago	24 (2.4)	2 (2.4)	
*Stroke (ischemic and hemorrhagic)* *(n = 1052 and n′ = 84)*			0.19
No	1006 (95.6)	79 (94.0)	
Yes < 10 years ago	12 (1.1)	3 (3.6)	
Yes ≥ 10 years ago	34 (3.3)	2 (2.4)	

Data are presented as number (percentage) of participants unless otherwise indicated. Bold values indicate statistically significant results with P-value < 0.05.

**Table 2:** Retinal vascular patterns derived from principal component analysis: factor loading for vascular factors in the Montrachet study (n = 1069).

	Factor 1 *	Factor 2 *	Factor 3 *
Six largest arterioles in zone B †	−0.22	**0.86**	0.16
Six largest veins in zone B	−0.18	**0.70**	0.05
Arteriole/vein ratio of zone B measurements	0.05	0.07	0.11
Six largest arterioles in zone C ‡	−0.25	**0.84**	0.16
Six largest veins in zone C	−0.22	**0.72**	0.11
Arteriole/vein ratio of zone C measurements	0.07	0.06	0.05
Fractal dimension—total—zone C	−**0.67**	0.02	0.24
Fractal dimension—arterioles—zone C	**−0.59**	0.05	0.24
Fractal dimension—venules—zone C	**−0.51**	-0.02	0.24
Zone C mean width, arteriole	0.25	**0.85**	0.08
Zone C SD, arteriole	**0.90**	0.14	0.15
Zone B mean width, arteriole	0.28	**0.84**	0.08
Zone B SD, arteriole	**0.86**	0.13	0.22
Simple tortuosity, arteriole	0.01	0.12	**0.71**
Curvature tortuosity, arteriole	0.08	0.11	**0.79**
Number of 1st branchings, arteriole	−0.43	0.13	0.22
Branching coefficient, arteriole	−0.04	-0.06	−0.13
Asymmetry ratio, arteriole	−0.15	-0.04	−0.05
Branching angle, arteriole (summary measurement from the first branch traced on the six largest arterioles)	−0.14	0.09	0.07
Angular asymmetry, arteriole	−0.06	0.02	−0.02
Angle of 1st daughter	−0.11	0.08	0.08
Angle of 2nd daughter	−0.13	0.08	0.04
Junctional exponent deviation for arterioles	−0.07	0.14	0.10
Length/diameter ratio, arteriole	−0.23	0.01	0.20
Zone C mean width, venule	0.25	**0.78**	−0.02
Zone C SD, venule	**0.91**	0.12	0.09
Zone B mean width, venule	0.26	**0.78**	−0.07
Zone B SD, venule	**0.85**	0.14	0.12
Simple tortuosity, venule	0.09	-0.03	**0.74**
Curvature tortuosity, venule	0.09	-0.01	**0.78**
Number of 1st branchings, venule	−0.36	0.12	0.20
Branching coefficient, venule	−0.07	0.08	−0.03
Asymmetry ratio, venule (or asymmetry factor)	−0.12	0.03	−0.05
Branching angle, venule (summary measurement from the first branch traced on the six largest venules)	−0.15	0.02	0.07
Angular asymmetry, venule	−0.08	0.01	0.02
Angle of 1st daughter	−0.06	0.01	0.05
Angle of 2nd daughter	−0.14	0.02	0.05
Junctional exponent deviation for venules	0.00	0.02	−0.08
Length/diameter ratio, venule	−0.14	0.01	0.20
Zone C mean width, vessels	0.29	**0.92**	0.03
Zone C SD, vessels	**0.93**	0.14	0.12
Zone B mean width, vessels	0.29	**0.92**	−0.01
Zone B SD, vessels	**0.90**	0.15	0.19
Simple tortuosity, vessels	0.06	0.07	**0.90**
Curvature tortuosity, vessels	0.11	0.07	**0.92**
Number of 1st branchings, vessels	−0.48	0.16	0.26
Branching coefficient, vessels	0.15	-0.03	−0.12
Asymmetry ratio (or asymmetry factor)	0.04	-0.12	−0.02
Branching angle, vessels	0.03	0.02	0.19
Angular asymmetry, vessels	0.03	0.00	0.03
Angle of 1st daughter, vessels	0.00	0.02	0.13
Angle of 2nd daughter, vessels	0.04	0.01	0.14
Junctional exponent deviation for all vessels	−0.10	0.11	0.00
Length/diameter ratio, vessels	−0.19	−0.02	0.20

Abbreviation: SD, standard deviation

*Factor 1: *Sparse vascular network pattern*, Factor 2: *Increased vessel caliber pattern*, Factor 3: *Increased tortuosity pattern*

† Zone B: 1 optic disc diameter

‡ Zone C: 2 optic disc diameter Bold values indicate values > 0.50

**Table 3:** Main vascular features of the Montrachet study participants by tertiles of retinal vascular patterns.

Sparse vascular network pattern	Fractal dimension (no unit)	Median	Q1	Q3
Poor fit		1.44	1.41	1.46
Intermediate fit		1.40	1.37	1.42
Good fit		1.33	1.29	1.38
**Increased vessel caliber pattern**	Vessel caliber (μm)			
Poor fit		126.51	120.16	131.68
Intermediate fit		138.75	134.16	143.57
Good fit		149.74	144.19	158.02
**Increased vessel tortuosity pattern**	Vessel tortuosity (no unit)			
Poor fit		1.08	1.08	1.09
Intermediate fit		1.09	1.09	1.10
Good fit		1.11	1.10	1.12

**Table 4:** Baseline characteristics of the Montrachet study participants by tertiles of retinal vascular patterns.

	Sparse vascular network pattern *Decreased fractal dimension and increased standard deviation for arteriole and venule width*				Increased vessel caliber pattern *Large arteriole and venule diameter and width*			
Tertiles	1st	2nd	3rd		1st	2nd	3rd	
Fit*	Poor	Intermediate	Good	P-value	Poor	Intermediate	Good	P-value
**Age, mean ± SD, y** (n = 1069)	79.7 ± 3.8	80.1 ± 3.8	80.3 ± 3.9	0.12	80.7 ± 4.0	79.7 ± 3.7	79.8 ± 3.7	**0.002**
**Sex** (n = 1069)				**<0.001**				0.42
Males	28.1	30.7	41.2		35.9	32.2	31.9	
Females	36.6	34.5	28.9		31.9	33.9	34.2	
**Education** (n = 1064)				0.51				0.30
Primary education	31.7	36.6	31.7		35.9	33.1	31.0	
Lower secondary education	35.2	32.5	32.3		31.9	33.4	34.7	
Upper secondary education	28.5	36.0	35.5		40.2	24.9	29.9	
Tertiary education	35.6	29.5	34.9		29.5	36.1	34.4	
**Smoking status** (n = 985)				**0.02**				0.69
Nonsmokers	36.1	33.2	30.7		33.0	34.2	32.8	
Smokers	28.8	33.0	38.3		33.8	33.5	32.7	
**Treatment for systemic hypertension** (n = 1014)				**0.05**				0.40
No	33.8	37.6	28.6		30.9	35.3	33.8	
Yes	33.7	31.0	35.3		34.7	31.7	33.5	
**Systolic blood pressure, mean in the 3C study ± SD, mmHg**	138.9 ± 16.8	138.9 ± 16.5	142.8 ± 16.9	**0.007**	140.7 ± 16.3	140.2 ± 17.1	139.7 ± 17.2	0.46
**Body mass index, mean in the 3C study ± SD, kg/m**^**2**^ (n = 1066)	25.2 ± 3.5	25.4 ± 3.5	25.8 ± 3.7	**0.03**	25.3 ± 3.6	25.5 ± 3.5	25.6 ± 3.6	0.32
**Hypoglycemic treatment** (n = 957)				**0.03**				0.79
No	34.9	33.9	31.2		33.4	32.4	34.2	
Yes	26.5	30.6	43.0		31.4	35.5	33.1	
**Cholesterol-lowering treatment** (n = 1021)				0.74				0.30
No	34.8	33.3	31.8		38.3	31.2	30.5	
Yes	32.8	33.1	34.1		31.8	33.5	34.6	
**Medical history** (n = 1004)				0.76				0.10
No vascular event	33.6	33.4	33.0		33.9	33.8	32.3	
MACCE	32.3	31.7	36.0		29.2	29.8	41.0	

Abbreviation: MACCE: major adverse cardiovascular and cerebrovascular events. Data are presented as number (percentage) of participants unless otherwise indicated.

*Fit: distance between the patient and the component.

Bold values indicate statistically significant results with P-value < 0.05.

**Table 5:** Baseline characteristics of the Montrachet study participants by tertiles of retinal vascular patterns.

	Increased vessel tortuosity pattern *Increased tortuosity for both arterioles and venules*			
Tertiles	1^st^	2nd	3rd	
Fit*	Poor	Intermediate	Good	P-value
**Age, mean ± SD, y** (n = 1069)	80.5 ± 3.8	79.9 ± 3.8	79.7 ± 3.9	**0.008**
**Sex** (n = 1069)				0.62
Males	35.3	31.7	33.0	
Females	32.6	34.1	33.3	
**Education** (n = 1064)				0.30
Primary education	35.9	33.1	31.0	
Lower secondary education	31.9	33.1	31.0	
Upper secondary education	40.2	29.9	29.9	
Tertiary education	29.5	36.1	34.4	
**Smoking status** (n = 985)				0.97
Nonsmokers	33.5	33.0	33.5	
Smokers	33.8	33.5	32.7	
**Treatment for systemic hypertension** (n = 1014)				0.99
No	33.2	33.8	33.0	
Yes	33.4	33.4	33.2	
**Systolic blood pressure, mean in the 3C study ± SD, mmHg**	140.2 ± 16.3	139.9 ± 16.7	140.5 ± 17.6	0.97
**Body mass index, mean in the 3C study ± SD, kg/m**^**2**^ (n = 1066)	25.7 ± 3.6	25.1 ± 3.5	25.3 ± 3.5	0.20
**Hypoglycemic treatment** (n = 957)				0.98
No	33.6	33.5	32.9	
Yes	33.1	33.1	33.9	
**Cholesterol-lowering treatment** (n = 1021)				0.43
No	35.8	31.5	32.7	
Yes	32.3	33.9	33.8	
**Medical history** (n = 1004)				0.10
No vascular event	34.7	32.6	32.7	
MACCE	26.1	37.9	36.0	

Abbreviation: MACCE: major adverse cardiovascular and cerebrovascular events. Data are presented as number (percentage) of participants unless otherwise indicated.

*Fit: distance between the patient and the component.

Bold values indicate statistically significant results with P-value < 0.05.

In bivariate analyses, subjects with a good fit with the *Sparse vascular network pattern* were more likely to be male, smokers, with a higher mean systolic blood pressure, a BMI >25 kg/m^2^ and they were more prone to be treated for diabetes mellitus. A good fit with the *Increased vessel caliber pattern* was associated with a slightly younger age (P = 0.002). Subjects with an *Increased tortuosity pattern* were also significantly slightly younger (P = 0.008) (Table 3).

In the multivariate polytomous regression analysis, we systematically adjusted for age, sex and education level (Table 6). Given the low number of subjects with missing data concerning their education level (n = 5) and their BMI (n = 3), we excluded those individuals from further analyses. No interaction between treated systemic hypertension and diabetes was found. Participants with hypoglycemic treatment were more likely to display a *Sparse vascular network pattern* than subjects without such treatment (odds ratio, [OR] good vs poor fit, 1.68; 95% CI, 1.04–2.72; P = 0.04). Individuals with MACCE before their inclusion in the Montrachet study had increased retinal vessel caliber compared to patients with no vascular event (OR good vs poor fit, 1.67; 95% CI,1.02–2.70; P = 0.04). In addition, individuals with MACCE tended to have increased vessel tortuosity (OR good vs poor fit, 1.62; 95% CI, 0.98–2.70; P = 0.06).

**Table 6:** Multivariate polytomous regression analysis of retinal vascular pattern and cardiovascular background in theMontrachet study (n = 1064).

	Sparse vascular network pattern *Decreased fractal dimension and increased standard deviation for arteriole and venule width*				Increased vessel caliber pattern *Large arteriole and venule diameter and width*				Increased vessel tortuosity pattern *Increased tortuosity for both arterioles and venules*			
Fit	Intermediate vs poor		Good vs poor		Intermediate vs poor		Good vs poor		Intermediate vs poor		Good vs poor	
	OR	95% CI	OR	95% CI	OR	95% CI	OR	95% CI	OR	95% CI	OR	95% CI
**Age**	1.03	0.99–1.07	**1.04**	**1.00–1.09**	**0.93**	**0.89–0.97**	**0.94**	**0.91–0.98**†	**0.96**	**0.92–0.99**	**0.95**	**0.91–0.99**†
**Sex**												
Males vs females	1.14	0.80–1.64	**1.71**	**1.20–2.44**†	0.83	0.61–1.14	0.80	0.58–1.09	0.77	0.56–1.06	0.83	0.61–1.13
**Education**												
Lower secondary vs primary	0.80	0.50–1.26	0.90	0.56–1.45	0.79	0.49–1.27	0.86	0.54–1.39	1.13	0.71–1.78	1.24	0.78–1.97
Upper secondary vs primary	1.04	0.62–1.77	1.16	0.67–2.00	0.79	0.47–1.34	**0.55**	**0.32–0.94**	0.82	0.49–1.37	0.88	0.52–1.47
Tertiary vs primary	0.67	0.40–1.13	0.83	0.49–1.41	0.67	0.40–1.13	0.69	0.41–1.16	1.38	0.82–2.30	1.37	0.81–2.31
**Smoking status**												
Smokers vs nonsmokers	1.21	0.85–1.75	1.35	0.94–1.94								
Missing vs nonsmokers	1.55	0.57–4.19	1.58	0.62–4.05								
**Hypoglycemic treatment**												
Yes vs no treatment	1.17	0.70–1.94	**1.68**	**1.04–2.72**								
Missing vs no treatment	0.73	0.31–1.75	1.03	0.47–2.30								
**Medical history at Montrachet inclusion**												
MACCE vs no vascular event					1.19	0.71–2.00	**1.67**	**1.02–2.73**	1.63	0.98–2.70	1.62	0.98–2.70
Missing vs no vascular event					0.89	0.46–1.71	1.22	0.65–2.26	1.29	0.68–2.44	1.22	0.64–2.33

*Fit: distance between the patient and the component. Bold values indicate statistically significant results with P-value < 0.05. †Bold values with * indicate statistically significant results with P-value < 0.001.

Finally in participants with no MACCE history, we identified an association between subjects with the best fit to a *Sparse vascular network* and the highest Heart SCORE (OR ≥ 10% vs < 5%, 1.76; 95% CI, 1.08–2.25; P = 0.02) and a trend for the intermediate fit (OR 5–10% vs < 5%, 1.56; 95% CI, 1.03–2.98; P = 0.04) (Table 7).

**Table 7:** Multivariate polytomous regression analysis of Heart SCORE and vascular pattern in the Montrachet study (n = 873).

	Heart Score					
	5–10% vs < 5%			> 10% vs < 5%		
	OR	95% CI	P-value	OR	95% CI	P-value
**Sparse vascular network pattern**						
Intermediate fit vs poor fit	1.39	0.96–2.03	0.08	0.90	0.48–1.67	0.73
Good fit vs poor fit	**1.56**	**1.03–2.98**	**0.04**	**1.76**	**1.08–2.25**	**0.02**
**Age**	0.98	0.94–1.03	0.38	1.01	0.96–1.07	0.68
**Education**						
Lower secondary vs primary	1.34	0.80–2.23	0.26	1.21	0.59–2.47	0.60
Upper secondary vs primary	1.71	0.98–3.00	0.06	1.27	0.56–2.86	0.57
Tertiary vs primary	**2.63**	**1.54–4.50**	**<0.001**	1.39	0.63–3.09	0.41
**Body mass index**	**1.05**	**1.00–1.09**	**0.04**	**1.10**	**1.04–1.17**	**0.002**
**Hypoglycemic treatment**						
Yes vs no treatment	1.43	0.91–2.25	0.12	1.63	0.87–3.06	0.13
Missing vs no treatment	0.82	0.48–1.38	0.59	1.03	0.50–2.13	0.96

Bold P-values indicate statistically significant results.

## Discussion

Although there is a large body of literature on the association between single retinal vascular network features and systemic vascular history, to the best of our knowledge this is the first study to integrate all geometric retinal vascular characteristics in one analysis. The PCA allowed us to account for and summarize as best possible all quantitative information given by the SIVA program on retinal vascularization. Three independent vascular patterns were identified. The most relevant factor was the *Sparse vascular network pattern* due to its ability to account for most of the explained variance and its significant association with CVD past history and future 10-year risk of cardiovascular death. The *Sparse vascular network pattern* was mainly characterized by a lower fractal dimension. This factor was able to describe the structural branching and density of the vascular retinal network. It showed subtle changes in retinal microcirculation. The measurement of density and evaluation of vascular branching as a fractal dimension was a major step forward in retinal microvasculature description. Numerous studies have demonstrated the association between suboptimal retinal vascular network and cardiovascular history, which supports our findings [24, 25]. Although debated, other patterns such as vessel caliber could be influenced by the heart cycle and axial length of the eyeball; this emphasizes the increasing interest in the fractal dimension [26, 27]. Moreover, with greater variability in the vascular width, the *Sparse vascular network pattern* seems to be the best model for summarizing suboptimal microvascular architecture and the lack of optimal vascular design according to the Murray principle of minimum work [28]. In addition, the strong association between *Sparse vascular network pattern* and treated diabetes mellitus makes sense because diabetes is known to affect retinal microvasculature. Pericyte apoptosis and activation of the renin-angiotensin system are one of the leading early mechanisms of the impact of diabetes on retinal microvasculature, which probably explains decreased vascular density [29–31].

Although the association between the fractal dimension and cardiovascular risk factors is well documented, few studies have focused on a suboptimal retinal vascular network and cardiovascular mortality risk [32]. The Heart SCORE was validated in the European population and it is the current European CVD risk assessment model. It is still unknown why *Sparse vascular network pattern* is related to an increased cardiovascular mortality risk. We assumed that suboptimal retinal microvasculature reflects systemic vessel remodeling, which leads to hypertension, nephropathy, arteriosclerosis leading to increased cardiovascular mortality risk [33]. In the future, one could imagine that retinal microvasculature analysis with SIVA could be associated with other features in the Heart SCORE assessment model and account for an incremental prognostic value of this score. If these findings are confirmed by future studies, it could be assumed that patients with a *Sparse retinal vascular network*, measured with retinal analysis software, could benefit from an earlier and rigorous cardiovascular risk factor monitoring.

We did not find any relationship between vessel caliber or vessel tortuosity and high blood pressure. One of the reasons postulated may be that the profile of the second and third patterns–*Increased vessel caliber* and *Increased vessel tortuosity*–include both venules and arterioles in the same direction. In fact, history and risk of developing systemic hypertension are associated with narrower retinal arteriolar diameter and wider venular diameter [34]. Moreover, Cheung et al. showed that less tortuous arterioles (lower retinal arteriolar tortuosity value) and more tortuous venules (higher retinal venular tortuosity value) were independently associated with higher blood pressure [18]. Since we did not succeed in discriminating venules and arterioles with the PCA analysis, the second and the third patterns remain difficult to interpret as well as their relationship with previous history of MACCE.

The strengths of this study include a large population-based study, a wide range of systemic medical features and a 10-year data collection through the 3C study’s medical information.

We acknowledge several limitations to this study. First, Montrachet participants are urban volunteers. These subjects follow a healthy lifestyle and they have benefited from steady access to medical care. Therefore, the prevalence of cardiovascular events was very low in the population and we suspect that cardiovascular risk factors were well monitored. Second, these findings based on a Caucasian European population cannot be extrapolated to other parts of the world and other ethnicities. Third, we did not perform carotid Doppler ultrasonography at the time that fundus photographs were taken. Liao et al. already showed that ipsilateral carotid artery stiffness was associated with generalized narrowing of the retinal arterioles [35]. Fourth, cardiovascular history was collected from self-declarations by participants, which introduced a reporting bias. Fifth, this exploratory cross-sectional study only enhanced a potential association between fatal CVD and the retinal vascular pattern. This has to be confirmed by a longitudinal study to validate our findings. Finally, we are planning to conduct future studies with these vascular patterns adding potential confounders such as axial length, dietary factors and exercise levels.

In summary, this study strengthens the idea that retinal vascular network analysis is a promising tool to assess systemic vascular status and possible cardiovascular mortality. This study tends to confirm the appeal of the fractal dimension and highlights the *Sparse vascular network pattern* as the most appropriate feature to assess the vascular aging process. Moreover, this *Sparse vascular network pattern* is associated with a higher fatal CVD risk within the next 10 years. Further prospective studies should be implemented to validate retinal vessel analysis as a marker for cardiovascular morbidity and mortality.
